# Syndrome Differentiation of Diabetes by the Traditional Chinese Medicine according to Evidence-Based Medicine and Expert Consensus Opinion

**DOI:** 10.1155/2014/492193

**Published:** 2014-07-14

**Authors:** Jing Guo, Hongdong Chen, Jun Song, Jia Wang, Linhua Zhao, Xiaolin Tong

**Affiliations:** Guang'anmen Hospital, China Academy of Chinese Medical Sciences, Beijing 100053, China

## Abstract

In Chinese medicine, diabetes belongs to the category of “Xiaoke disease (disease with symptoms of frequent drinking and urination)”; in the traditional sense, its pathogenesis is “Yin deficiency and dryness-heat.” However, over time, changes in the social environment and lifestyle have also changed the use of traditional Chinese medicine (TCM) in diabetes. In this study, we performed diabetes syndrome differentiation using TCM according to evidence-based medicine and expert consensus opinion.

## 1. Introduction

Diabetes mellitus (DM) is often caused by the consumption of a high fat and calorie diet. It has a high prevalence and can often lead to complications that seriously affect the quality of life of sufferers. In 2013, according to the latest statistics of the International Diabetes Federation (IDF), the global prevalence of diabetes among adults aged 20–79 was 8.3%. The total number of patients with diabetes worldwide was estimated to be 382 million, which was predicted to rise to nearly 592 million by 2035. Therefore, research on the prevention and treatment of diabetes is critical and represents a great challenge for the medical profession.

In recent years, Chinese medicine has made great progress toward the prevention and the treatment of diabetes, and its curative effects have been widely recognized. The clinical effects of the kai-yu-jiang-zhuo decoction [1] are the same as metformin; therefore, the kai-yu-jiang-zhuo decoction could be recommended clinically. In one study  [2] assessing the ability of the Chinese herbal medicine Tianqi to reduce progression from impaired glucose tolerance to diabetes, there was a significant difference in the number of subjects who had normal glucose tolerance at the end of the study between the Tianqi (63.13%) and placebo groups, respectively (46.60%). Cox's proportional hazards model analysis demonstrated that Tianqi reduced the risk of diabetes by 32.1% compared with placebo. Similarly, tang-min-ling pills [3] could effectively reduce glycosylated hemoglobin levels and fasting blood glucose (FBG) and improve islet *β* cell function. As the main ingredient in Coptis, Berberine [4, 5] also has good hypoglycemic effects and improves insulin resistance.

Treatment based on syndrome differentiation is the basic principle of illness and treatment in traditional Chinese medicine (TCM). To improve symptoms, individualized treatment plans are more scientific and superior. A previous study [6] suggested that the Chinese medicinal dialectical treatment of patients with diabetes was highly effective. In addition, dialectical treatment is relatively flexible, causes less adverse reactions, and is safe and reliable. These characteristics can effectively improve the quality of life of diabetic patients and are worth assessing clinically. However, due to the inadequate international understanding of the culture of TCM, syndrome differentiation is often avoided or disregarded, which reduces the potential benefits of TCM.

In TCM [7], the diagnoses and treatment of Xiaoke disease were traditionally based on “three excessive and one loss” symptoms, excessive fluid drinking, excessive food-consumption, excessive urination, and weight loss and its core pathogenesis is “Yin deficiency and dryness-heat.” However, 80% of type 2 diabetes patients do not have these three typical symptoms, so are very different form Xiaoke. Approximately 85% of type 2 diabetes patients are overweight or obese, suggesting that these obese diabetes patients form the majority of the diabetic population. In addition, compared with prior living environments, the modern diet has changed significantly, which has resulted in a significant increase in the diabetic population. Fewer individuals are thin, and an increasing number of people are obese. Thus, physicians gradually realized that the typical SanXiao symptoms (three types of diabetes that, resp., involving the upper-jiao, middle-jiao, and the lower warmer) usually develop later during the pathogenesis of diabetes, and so most people with diabetes do not present with these symptoms. The traditional TCM theory of Yin deficiency and dryness-heat is more difficult to obtain a satisfactory curative effect during the treatment of diabetes; therefore, novel theories have been proposed. Diabetes symptoms are complex, and physicians do not have unified opinions regarding the pattern identification based the syndrome differentiation of diabetes. Guidelines of Prevention and Treatment of Diabetes by TCM, the first guidelines for diabetes, which was issued in 2007 as a project funded by the State Administration of Traditional Chinese Medicine, promoted the diagnosis and treatment of diabetes and its complications. The guidelines were proposed and revised repeatedly by members of the standing committee of diabetes branch of the China Association of Chinese medicine and were confirmed by the domestic renowned diabetes experts Linlan, Zhang Farong, Li DeLin, and Cheng Yichun. With the unification of TCM and Western medicine terminologies related to diabetes and its complications, diabetes-related terminology gradually became normalized and standardized. In this study, we perform diabetes syndrome differentiation of TCM based on the guidelines (2007) and evidence-based medicine.

## 2. Syndrome Differentiation

“TCM syndrome differentiation and evaluation standard of DM”, “Guidelines of Prevention and Treatment of Diabetes by TCM, 2007,” and “DM treatment using integrated traditional Chinese and Western medicinal therapy” have all been proposed for the TCM-mediated differentiation of the clinical stages of DM [8, 9]. In one study [10], stagnancy, heat, deficiency, and damage were thought to be the four stages of diabetes, and collateral damage existed through the course of the disease even before the diagnosis. The collateral damage in different degrees could be defined as “collateral qi obstruction, collateral qi stagnation, collateral blockage, and collateral damage.” The study also suggested that the TCM-mediated differentiation with the clinical stages of DM is indispensable. Therefore, we performed syndrome differentiation based on the clinical stages of diabetes.

### 2.1. Syndrome Differentiation of DM

#### 2.1.1. QI Stagnation due to Liver Depression

According to modern medical research [11], patients with diabetes often exhibit aggravated emotional tension, which is consistent with the theory of TCM that negative emotions could lead to diseases. Liver depression could lead to qi stagnation and result in some emotional symptoms. This was the first stage of diabetes, and the characteristic was stagnancy. Therefore, soothing the liver and adjusting qi are the main therapeutic principles. This type of diabetic patients shows some emotional symptoms such as depressed mood, like frequent sighing, nervousness, distention, and fullness in the chest and rib-side. The patients usually have a pale tongue with thin white moss and a stringy pulse. Modified Xiao Chaihu decoction, a classic Chinese ancient prescription, was commonly used to treat this type of diabetic patients; some herbs like Bupleurum, Scutellaria baicalensis, Pinellia ternata, and Ginseng were included in this decoction [12–15].

#### 2.1.2. Liver and Stomach Heat Stagnation

A symptom analysis of 2518 obese patients with type 2 diabetes [16] demonstrated that there were 1332 cases of liver and stomach heat stagnation syndrome, accounting for 52.9% of all the cases, suggesting that it was an important type in diabetes syndrome differentiation. Liver and stomach heat stagnation belong to the stagnancy and heat stages of diabetes. The patients of this type showed some emotional and digestive symptoms such as irritability, distention and fullness in the chest and rib-side, drinking too much fluids and the production of increased urine, eating too much food, hunger, experiencing a bitter taste, dry mouth, and constipation. And patients usually have a red tongue, and a rapid and stringy pulse. Clearing stagnation-heat of liver and stomach is an important therapeutic principle for this type. A modified Da Chaihu decoction, one of classic Chinese ancient prescriptions recorded in Treatise on Cold Pathogenic and Miscellaneous Diseases, was used to treat such diabetic patients. Some Chinese herbs like Bupleurum, Chinese rhubarb, Scutellaria baicalensis, Citrus aurantium, Radix Paeoniae Rubra, and so forth were included in this formula [17, 18].

#### 2.1.3. Phlegm and Heat Stasis

A study by Gan et al. [19] showed that phlegm and heat stasis syndrome was a common type in the early and middle stages of diabetes and accounted for more ratios particularly in patients who smoked and drank alcohol. Zhou et al. [20] investigated 344 patients with type 2 diabetes and found that 101 cases (29.4%) belonged to phlegm and heat stasis syndrome. This syndrome often appears in the “heat” stage of diabetes, and the patients are relatively obese because in the theory of Chinese medicine, “obese people tend to have copious phlegm.” Patients with this type may have some symptoms such as abdominal obesity, a sense of chest suppression, abdominal distention, and dry mouth. They might also prefer cold drinks, drink much more fluids, and be irritable and have a bitter taste in their mouth as well as constipation. Patients also have a red and fat tongue with yellowish greasy moss, yellow urine, and a stringy and smooth pulse. Reducing heat and removing phlegm is the therapeutic principle in this syndrome, and a modified Xiao Xianxiong decoction, a classic Chinese ancient prescription, is used to treat such diabetic patients. Some Chinese herbs like rhizoma coptidis, Pinellia ternata, snakegourd seed, and so forth are included in this formula [21–24].

#### 2.1.4. Excess Heat in the Stomach and Intestine

Both Tong and Wang's studies [16, 25] demonstrated that “*excess heat in the Stomach and intestine*” was one of main syndromes of diabetes. This syndrome generally occurs in the diabetic middle stage or in the “heat” stage. In the middle stage of diabetes, patients eat large amounts of food, which stagnate and form heat in the stomach and intestine. As such, its principal symptoms are abdominal fullness and distention, constipation, a bitter taste and dry mouth, halitosis, thirst with a desire for cool water, drinking and eating too much, and hunger. Patients usually have a red tongue with yellow moss and a rapid strong pulse. To remove the heat, a modified Dahuang Huanglian Xiexin Decoction is regarded as the main prescription which includes Chinese rhubarb and rhizoma coptidis and so forth [25–27].

#### 2.1.5. Intestinal Damp and Heat Syndromes

According to the findings of Zhao et al. [28] in a study using classical prescriptions to treat different diseases, the Gegen Qinlian decoction could be used to treat type 2 diabetes with concurrent intestinal damp and heat syndromes, with a good clinical efficacy. An additional study [29] showed that Gegen Qinlian decoction could significantly improve the intestinal damp and heat syndrome scores of patients with type 2 diabetes and could effectively control blood glucose with a success rate of 88.6%. Another study [30] revealed that the morbidity of type 2 diabetes with damp and heat syndrome was 30.7% and that the location of disease was in Fu-organs. In addition, this syndrome has unique features. The intestinal damp and heat syndromes always appear in the diabetic middle stage or during the heat stage. Its principal symptoms are thirst with no desire to drink, hunger with no desire to eat, a bitter taste, a sticky and greasy sensation in the mouth, and abdominal distention. Patients also show a red tongue with yellow and greasy moss and a slippery pulse. When damp and heat affect the large intestine, smelly greasy stools might also form. To reinforce the spleen and stomach and remove the heat and dampness, a modified Gegen Qinlian decoction, one of classic Chinese ancient prescriptions, is used to treat such diabetic patients. Some Chinese herbs like kudzuvine root, Scutellaria baicalensis, rhizoma coptidis, and so forth are included in this formula [29, 31].

#### 2.1.6. Deficiency of Body Liquid due to Excessive Heat Syndrome

A study by Gan and Chen [32] suggested that excessive heat injuring liquid syndrome was a principle syndrome of diabetes. Consistent with this, Zhang et al. [33] reached the same conclusion after investigating 1490 cases of type 2 diabetes using clinical syndrome differentiation. The deficiency of body liquids due to excessive heat syndrome is more commonly found in the diabetic middle-late stage or the heat and deficiency stages. Impacted by the fire and heat pathogens from the early and middle stages of diabetes, qi is consumed and liquids are injured gradually. As such, its principle symptoms are dry throat and mouth, thirst with a desire for cool water, overeating and hunger, frequent micturition volume, irritability, bitter taste, red urine, and constipation. Patients also commonly have a red tongue with yellow fur and a rapid pulse. To sooth the heat and promote fluid production, a modified Xiaoke Wan or Baihu Tang, belonging to classic Chinese ancient prescriptions recorded in Treatise on Cold Pathogenic and Miscellaneous Diseases, is used to treat such diabetic patients. Some Chinese herbs like Gypsum, Rhizoma Anemarrhenae, Liquiritia Glycyrrhiza, and so forth are included in this formula [34–36].

#### 2.1.7. Dual Deficiency of Qi and Yin

Based on her 40 years of clinical experience, Lin and Ni [37] proposed a theory called III-type differentiation, which proposes that the dual deficiency of qi and yin syndrome was one of basic syndromes of diabetes. Many other professors, including Xu et al. [38], Mao et al. [39], and Li et al. [40] also concluded that the dual deficiency of qi and yin syndrome was a common syndrome of diabetes. The dual deficiency of qi and yin syndrome occurs in the late diabetic or the deficiency stage. The fire and heat pathogens further dissipate the primordial qi of zang-fu organs, and then the generalized qi is consumed. In addition, fire and heat pathogens scorch liquids and damage yin. Therefore, the main symptoms are dry throat and mouth, thirst with a large intake of fluid, fatigued spirit and lack of strength, shortness of breath and reluctance to speak, emaciation of the body, limp aching lumbus and knees, spontaneous and night sweats, feeling palm and arch fever, upset, palpitations, insomnia, a red tongue with scant liquids and thin white dry tongue fur, and a fine rapid pulse. Boosting qi and nourish yin is one of the important therapeutic principle in this type, and a modified Shengmai Yin decoction, a classic Chinese ancient prescription, is used to treat such diabetic patients. Some Chinese herbs like, Ophiopogon japonicas, Schisandra chinensis, ginseng, and so forth are included in this formula [41, 42].

### 2.2. The Stage of Diabetes Complications

During the diabetic complications stages, treatment requires the combination of disease and syndrome differentiation due to its complexity. In general, the deficiency is increasingly aggravating, and so qi-blood-liquid deficiency and the function of internal organs decline. It belongs to late stage of diabetes; liver and kidney insufficiency and deficiency in both yin and yang are its endpoints. The main syndromes in this stage include insufficiency of the liver and kidney and detrimental yin and yang [10, 15].

#### 2.2.1. Insufficiency of the Liver and Kidney Syndrome

The main symptoms are urinary frequency, turbid unctuous, and limp aching and lumbus and knees, which are accompanied by additional symptoms including blurred vision, dizziness, tinnitus, red tongue with some fur, and a fine rapid pulse. Modified Qiju Dihuang Wan, which includes the fruit of Chinese wolfberry, chrysanthemum, Chinese yam, and Cornus officinalis, is used to treat these diabetic patients by enriching the liver and kidney with essence and increasing blood supply [43–45].

#### 2.2.2. Dual Deficiency of Yin and Yang Syndrome

Patients with this syndrome exhibit symptoms including urinary and nocturia frequency, which can be accompanied by feeling palms and arches fever, being upset, dry throat or mouth, limp aching lumbus and knees, fear of the cold, icy cold limbs, and a forceless fine sunken pulse. Enriching yin and supplying yang is an important therapeutic principle; modified Jingui Shenqi decoction, which includes the Chinese herbs adhesive rehmannia dried root, Chinese yam, Fructus Corni, cassia twig, and monkshood, is commonly used to treat these diabetic patients   [46–49].

Studies [50] have shown that the characteristic pathophysiological mechanism of chronic diabetic complications is root deficiency and tip excess. Deficiency and static blood occur throughout several complications. The dual deficiency of qi and yin, phlegm turbidity, and static blood obstructing the network vessels form the common pathological basis of diabetic chronic complications. Studies [51] using the collateral disease theory have determined that static blood obstructing the collateries is the pathological basis for diabetic microangiopathy; therefore, treatment should be aimed to promote blood circulation and remove obstruction in vessels throughout the whole process. A large number of clinical observations and scientific studies have confirmed that capillaries can be protected and diabetic microvascular complications prevented and treated using drugs that accelerate blood flow during early-mid diabetes.

Generally, treatments for diabetic complications should target phlegm, static blood, and additional pathological factors, except for insufficiency of the liver and kidney and dual deficiency of qi and yin, for more comprehensive and thorough evidence-based medicine.

## 3. Diabetic Patterns and Correlation Indices

### 3.1. Pattern Types according to the Function of Insulin

Studies [52–54] have demonstrated that diabetic syndrome differentiation is closely correlated with insulin function, which provides an objective basis for syndrome differentiation using TCM. During the pathogenesis of diabetes, islet *β*-cell function changes from the compensatory period to the mildly decompensated period, severe decompensated period, and decompensated with structure damage, which correspond to the different stages of diabetes. Concurrently, during early diabetes, qI stagnation occurs due to liver depression and other syndromes. In the middle stage, intestinal dampness-heat and other syndromes occur. Finally in the middle and late stages the dual deficiency of qi and yin and the dual deficiency of yin and yang and other syndromes are present; islet *β*-cells are increasingly damaged. As a result, insulin secretion gradually declines as symptoms evolve. Varying degrees of insulin resistance occur with different symptoms and commonly first increase and then decline as symptoms evolve. This might be because, in the late stages of diabetes whereas the number of islet *β*-cells gradually decline and the number of insulin receptors on target organs relative increases.

### 3.2. Diabetic Patterns of Inflammatory Markers

Studies [55] have shown that CRP, IL-6, and other cytokines mediate insulin metabolic pathways, weaken insulin receptor signal transduction, induce disorders of glucose metabolism, and stimulate type 2 diabetes. Additional studies   [56, 57] assessing the long-term risk factors for type 2 diabetes revealed that the levels of CRP, IL-6, IL-8, and TNF-*α* were higher in diabetic populations than the normal population. These indexes levels increase gradually as syndromes evolve; in particular, the levels of those indexes of the dual deficiency of yin and yang symptom are higher than those of any other symptoms. Consequently, IL-6, IL-8, and TNF-*α* can be used as objective indicators during the TCM-mediated syndrome differentiation of DM [58].

### 3.3. Diabetic Patterns of Biochemical Indicators

Fasting plasma glucose, postprandial blood sugar, and glycosylated hemoglobin were significantly higher in diabetic populations than normal individuals, but there were no significant differences among different syndromes [59, 60].

When combined with high uric acid (UA) hematic disease, the UA levels of type 2 diabetic patients with dual deficiency yin and yang syndrome were higher than those of other syndromes, which suggested that the dual deficiency of yin and yang might be a factor leading to elevated blood UA levels [61].

Compared with normal individuals, obvious lipid metabolic disorders occur in patients with type 2 diabetes, including increased triglycerides (TG), total cholesterol (TC), low density lipoprotein (LDL), and decreased high density lipoprotein (HDL). Phlegm and heat stasis changed the most with an increased severity of symptoms. As the course of the disease lengthened and TC and LDL-C increased, the lipid metabolism disorders of phlegm and heat stasis symptom became most serious [62, 63].

### 3.4. Diabetic Pattern Types of Hemorheology

Studies [58, 64] have shown that significant changes occur between the hemorheological performances of normal and type 2 diabetes populations. The levels of blood specific viscosity, fibrinogen, hematocrit, erythrocyte sedimentation rate (ESR), and other indicators of dual deficiency of qi and yin symptom were significantly higher than those of normal populations. This suggested that the occurrence of diabetes was associated with increased blood viscosity, and that the dual deficiency of qi and yin was the pathological basis for the change of blood rheology.

### 3.5. Diabetic Patterns of Related Genes

Studies [65] on the relationship between syndrome differentiation of diabetes by TCM and the level of calcitonin gene related peptide (CGRP) showed that the levels of CGRP of every syndrome of diabetes are significantly lower than normal people, and the dual deficiency of yin and yang has the lowest level of CGRP. Another study [66] to explore the relation between Chinese medicine syndrome and the gene polymorphism of peroxisome proliferator-activated receptor delta (PPARD)-87C>T, the genotype frequencies of T/C and C/C at PPARD-87C>T are higher in the dual deficiency of yin and yang syndrome in newly diagnosed type 2 diabetes patient. Newly diagnosed type 2 diabetes patients with these alleles have higher levels of plasma glucose and lipids. This suggests that the PPARD-87C>T polymorphism might be a factor that affects the progression of type 2 diabetes.

An additional study [67] on the substance of dual deficiency of qi and yin from the molecular level used a diabetes gene array (Superarray Bioscience) containing 96 key diabetes-related genes to identify that 43 differentially expressed genes between normal and diabetic dual deficiency of qi and yin patients. Compared with the gene levels of normal people, 35 were upregulated and eight were downregulated. And further RT-polymerase chain reaction (RT-PCR) and Western-blotting measurement confirmed Fork head box C2 (FOXC2) and IRS-2 mRNA are specific genes of diabetic patients with dual deficiency of qi and yin syndrome.

## 4. Summary

The TCM emphasizes individualized treatment and pay attention to yin-yang balance and a holistic approach. Deep understanding of diabetes' clinical manifestation and symptom differentiation from different aspects are very important to improve the clinical effects, such as uncomfortable symptoms, blood glucose, blood lipids, and diabetes complications. Though symptom differentiation of TCM is relatively difficult to understand, with the development of modern medicine and researcher's efforts on it, people will get familiar with it and make TCM play an important role in diabetic fields.
